# Working Memory and Transcranial-Alternating Current Stimulation—State of the Art: Findings, Missing, and Challenges

**DOI:** 10.3389/fpsyg.2022.822545

**Published:** 2022-02-14

**Authors:** Wiam Al Qasem, Mohammed Abubaker, Eugen Kvašňák

**Affiliations:** Department of Medical Biophysics and Medical Informatics, Third Faculty of Medicine, Charles University in Prague, Praha, Czechia

**Keywords:** transcranial alternating-current stimulation, working memory, brain oscillations, non-invasive brain stimulation, cognitive deficits

## Abstract

Working memory (WM) is a cognitive process that involves maintaining and manipulating information for a short period of time. WM is central to many cognitive processes and declines rapidly with age. Deficits in WM are seen in older adults and in patients with dementia, schizophrenia, major depression, mild cognitive impairment, Alzheimer’s disease, etc. The frontal, parietal, and occipital cortices are significantly involved in WM processing and all brain oscillations are implicated in tackling WM tasks, particularly theta and gamma bands. The theta/gamma neural code hypothesis assumes that retained memory items are recorded via theta-nested gamma cycles. Neuronal oscillations can be manipulated by sensory, invasive- and non-invasive brain stimulations. Transcranial alternating-current stimulation (tACS) and repetitive transcranial magnetic stimulation (rTMS) are frequency-tuned non-invasive brain stimulation (NIBS) techniques that have been used to entrain endogenous oscillations in a frequency-specific manner. Compared to rTMS, tACS demonstrates superior cost, tolerability, portability, and safety profile, making it an attractive potential tool for improving cognitive performance. Although cognitive research with tACS is still in its infancy compared to rTMS, a number of studies have shown a promising WM enhancement effect, especially in the elderly and patients with cognitive deficits. This review focuses on the various methods and outcomes of tACS on WM in healthy and unhealthy human adults and highlights the established findings, unknowns, challenges, and perspectives important for translating laboratory tACS into realistic clinical settings. This will allow researchers to identify gaps in the literature and develop frequency-tuned tACS protocols with promising safety and efficacy outcomes. Therefore, research efforts in this direction should help to consider frequency-tuned tACS as a non-pharmacological tool of cognitive rehabilitation in physiological aging and patients with cognitive deficits.

## Introduction to Brain Oscillations and Working Memory

### Working Memory

Working memory (WM) is a cognitive process that involves maintaining and processing information for a short period of time (Baddeley, 2012). WM is highly involved in goal-directed behavior (Chai et al., 2018), and was described by Baddeley and Hitch’s multicomponent model before the advent of competing models (Baddeley and Hitch, 1974; Daneman and Carpenter, 1980; Engle, 2002; Barrouillet et al., 2004, 2009; Cowan, 2008). Among the competing models of WM: Daneman and Carpenter’s (1980) model, which showed that the capacity of WM is reflected by the reading span task and comprehensive tests; the time-based resource sharing model proposed by several researchers, which showed that the capacity of WM is a product of attention (Barrouillet et al., 2004, 2009). Details of other relevant WM models can be found in the review article by D’Esposito and Postle (2015). The multicomponent model initially consisted of three subcomponents, namely the phonological loop (verbal WM), the central executive, and the visuospatial sketchpad (visuospatial WM) (Baddeley and Hitch, 1974; Baars and Franklin, 2003; Cowan, 2008; Kim et al., 2015). Then, episodic memory was added to the three main subcomponents of the WM model (Baddeley, 2000; RepovŠ and Baddeley, 2006). WM consists of three main steps: (1) encoding (memory items); (2) maintenance and manipulation of information; and (3) retrieval (recalling of the information) (D’Esposito, 2007; Baddeley, 2012).

The occipital and fronto-parietal cortices, particularly the dorsolateral prefrontal cortex (DLPFC), the anterior cingulate cortex (ACC) and the parietal cortex, are thought to be highly involved in the processing of WM (Osaka et al., 2003; Kim et al., 2015; Chai et al., 2018). This involvement is clearly evident in behavioral studies in which non-invasive stimulation of DLPFC (Kehler et al., 2020), ACC (Turi et al., 2020), and parietal cortex (Wolinski et al., 2018) influences the outcomes of WM tasks. The DLPFC plays an important role in maintaining information by retrieving stored information, inhibiting irrelevant stimuli, updating information, and top-down control of memory processes (Osaka et al., 2003; Moore et al., 2013; Vartanian et al., 2013; Kim et al., 2015). ACC acts as an “attention controller” assessing the need to adapt the information received based on task requirements (Osaka et al., 2003). The parietal cortex is thought to be the “workspace” for sensory and perceptual processing (Owen et al., 2005; Andersen and Cui, 2009).

Working memory is important for supporting higher cognitive processes such as learning, planning, mathematical skills, reasoning, etc. Therefore, WM deficits underlie problems in higher cognitive processes and developmental difficulties (Baddeley, 2003; Jeffries and Everatt, 2004; Raghubar et al., 2010; Logie, 2011). It is well known that neuroplasticity in the lateral prefrontal cortex decreases with age and memory deficits are apparent in the elderly (Hedden and Gabrieli, 2004; Ziaei et al., 2017; Goldsworthy et al., 2020; Haque et al., 2021). Moreover, older people are unable to optimally ignore task-irrelevant information (Borghini et al., 2018). However, aging is not the only factor affecting WM performance. WM deficits have been observed in a number of neurological and mental disorders such as schizophrenia, attention-deficit/hyperactivity disorder (ADHD), major depressive disorder (MDD), bipolar affective disorder, Alzheimer‘s disease, mild cognitive impairment (MCI) (Stegmayer et al., 2015; Maehler and Schuchardt, 2016; Grot et al., 2017; Le et al., 2017), and in patients with traumatic brain injury (Dunning et al., 2016; Phillips et al., 2017). Disturbances in the prefrontal cortex have been linked to WM deficits in patients with mood disorders particularly the orbital, and medial regions of prefrontal cortex, as well as DLPFC (Drevets, 2000; Barch et al., 2003).

### Brain Oscillations and Cross Frequency Coupling

Brain oscillations arise from the coordinated and synchronized activities of a large number of neuronal populations and are divided into five recognizable frequency bands: delta, theta, alpha, beta, and gamma, and they are involved in several functional processes in the brain (Cebolla and Cheron, 2019; Jensen et al., 2019). The anatomical locations and the functions in which the frequency bands are involved are listed in Table 1. Brain oscillations in different frequency bands have been associated with WM processes (Roux and Uhlhaas, 2014; Riddle et al., 2020b), particularly theta, gamma, and alpha bands. Theta oscillations play an essential role in the temporal organization of WM items, gamma oscillations affect the maintenance of WM information, while alpha oscillations play a role in inhibitory top-down control and the inhibition of task-irrelevant information (Klimesch et al., 2007; Roux and Uhlhaas, 2014).

**TABLE 1:** The anatomical locations and the functions of the frequency bands in the cortical structure.

Frequency band	Location	Function	References
Delta waves (0.5–4 Hz)	Cortex and thalamus	Slow-wave sleep, motivational and emotional processes, and inhibition of the sensory afferences during mental tasks	Harmony et al., 1996; Amzica and Steriade, 1998; Knyazev, 2007
Theta band (4–7 Hz)	Hippocampus, prefrontal and sensory cortices	Memory, long-range synchronization, synaptic plasticity, cognition, and behavior	Gevins et al., 1997; Seager et al., 2002; Vertes, 2005; Raghavachari et al., 2006; Tsujimoto et al., 2006; Sirota et al., 2008
Alpha band (8–12 Hz)	Hippocampus, sensory and motor cortices, and thalamus	Long-range synchronization, attention, inhibition, and consciousness	Klimesch, 1997; Pfurtscheller et al., 1997; Palva S. et al., 2005; Thut et al., 2011a
Beta band (13–30 Hz)	All cortical structure	Decision making, inhibition of motor planning, sensory processes, long-range synchronization, and attention	Kopell et al., 2000; Gross et al., 2004; Hong et al., 2008; Zhang et al., 2008
Gamma band (30–200 Hz)	All brain structures	Consciousness, perception, attention, memory, motor control, and synaptic plasticity	Tallon-Baudry et al., 1998; Fries et al., 2001; Uhlhaas et al., 2008

*Hz, hertz.*

Cross-frequency coupling (CFC) is the interaction between brain oscillations in different frequency bands (Jirsa and Müller, 2013; Sotero, 2016; Siebenhühner et al., 2020). There are several ways in which CFC can occur: Phase-to-phase, phase-to-frequency, power-to-power, and phase-to-power interactions (Jensen and Colgin, 2007). In phase-to-phase coupling: phase locking occurs between different frequency bands and their phase relationship remains constant (Schack et al., 2002; Palva J. M. et al., 2005); in phase-to-frequency coupling: the frequency of the faster oscillations is modulated by the phase of the slower oscillations (Jensen and Colgin, 2007); in power-to-power coupling: the power variation of the faster oscillations correlates with the power variations of the lower oscillations (Bruns and Eckhorn, 2004); in phase-to-power coupling: the power of the faster oscillations is modulated by the phase of the slower oscillations (Lakatos et al., 2005; Mormann et al., 2005; Canolty et al., 2006). One of the most well-known couplings associated with WM is theta/gamma phase-amplitude coupling (PAC). This coupling provides the neural substrate for the representation and maintenance of multiple WM items, which is called theta/gamma neural code. The hypothesis behind theta/gamma neural code assumes that retained memory items are recorded via theta-nested gamma cycles (Bahramisharif et al., 2018). Thus, theta/gamma PAC is considered to be the neurophysiological signature of WM (Axmacher et al., 2010; Lisman and Jensen, 2013). In this context, two models have been adopted; one model states that each gamma wave represents a single memory item and only a finite number of gamma waves can fit into a given theta cycle, which may limit the capacity of WM (Lisman and Idiart, 1995; Jensen and Lisman, 1996). The second model assumes that the entire gamma burst that fits into the theta cycle encodes a single memory item (Herman et al., 2013; Van Vugt et al., 2014). After a certain time, the memory items must be refreshed by the new gamma bursts. This reactivation occurs after a few theta cycles, which could explain the limited capacity of WM (Van Vugt et al., 2014). The causal relationship between memory and oscillations can be tested by modulating neuronal oscillations to causally alter behavior (Hanslmayr et al., 2019). Oscillatory entrainment refers to the modulation of neuronal oscillations and can be achieved via three main approaches: sensory entrainment (Adrian and Matthews, 1934; Romo and Salinas, 2003; Whittingstall and Logothetis, 2009; Ng et al., 2013), non-invasive electric/magnetic stimulation (Marshall et al., 2006; Sahlem et al., 2015; Bueno-Lopez et al., 2019), and invasive electrical stimulation (Borchers et al., 2012; Lee et al., 2013; Riva-Posse et al., 2018).

### Non-invasive Brain Stimulation

Non-invasive brain stimulation (NIBS) techniques refer to the use of transcranial stimulation to modulate brain excitability with an excellent safety profile when properly performed (Boes et al., 2018). They include two modalities: transcranial electrical stimulation (tES) and transcranial magnetic stimulation (TMS) (Fregni et al., 2005; Boes et al., 2018; Polanía et al., 2018). The best-known forms of tES are transcranial direct-current stimulation (tDCS), transcranial alternating-current stimulation (tACS), and transcranial random noise stimulation (tRNS). These methods induce reversible alterations in cortical activities (Paulus et al., 2016). In particular, tDCS uses static and weak direct current (Hanslmayr et al., 2019), while the electric current continuously changes its strength and reverses its direction, forming a sinusoidal wave in tACS (Marshall et al., 2006; Kuo and Nitsche, 2012; Sahlem et al., 2015). In tRNS, weak direct currents with random frequencies and amplitudes are applied (Widhalm and Rose, 2019). TMS briefly induces focal and strong currents in brain tissues by generating electromagnetic fields (Mishra et al., 2011). TMS can be administered as single pulse, pairs of pulses, or repeated pulses (Rossini et al., 2015; Valero-Cabré et al., 2017). Repetitive transcranial-magnetic stimulation (rTMS) refers to a sequence of TMS pulses administered at a specific intensity and frequency (Mishra et al., 2011).

Frequency-tuned NIBS is a recent approach in neuroscience in which the frequency of the externally applied entraining stimulus matches naturally occurring oscillations and is mainly used as a modulator of cognitive abilities (Vosskuhl et al., 2018). Moreover, entrainment of ongoing oscillations is strongest when the frequencies of the entraining stimulus match the endogenous frequencies (Ali et al., 2013; Vossen et al., 2015). Indeed, tACS and rTMS have been used to entrain endogenous brain oscillations in a frequency-specific manner and in turn cognitive functions (Balconi, 2013; Dayan et al., 2013; Fröhlich et al., 2015; Tavakoli and Yun, 2017). tACS can be administered online or offline; online tACS is applied during cognitive tasks, whereas offline tACS is administered immediately before or between tasks. The sustained brain activity following stimulation is referred to as the “aftereffect” (Veniero et al., 2015). Indeed, successful tACS produces long-lasting aftereffects, as has been shown with offline tACS (Pozdniakov et al., 2021). The aftereffect demonstrates changes in synaptic plasticity rather than entrainment *per se* (Bland and Sale, 2019). The prolonged aftereffects of tACS may be related to a phenomenon called spike-timing-dependent plasticity (STDP) (Elyamany et al., 2021). When input action potentials occur immediately before output action potentials, the synapse is strengthened, as suggested by the STDP phenomenon. Thus, if tACS is administered at a frequency close to the resonance frequency during stimulation, synapses in the stimulated region could be strengthened according to the STDP mechanism (Chang et al., 2021). Multiple sessions of tACS may consolidate the neuroplasticity effects, thus eliciting long-lasting effects (Chang et al., 2021; Elyamany et al., 2021). In general, endogenous oscillations are stimulated by tACS through several mechanisms: (1) Oscillatory entrainment, i.e., when tACS is applied at a frequency above that of an endogenous oscillation, the frequency of that oscillation is accelerated; (2) increasing endogenous oscillation amplitude by applying tACS at the same frequency as that of the intrinsic target; (3) cross-frequency coupling by applying predetermined stimulation frequencies, which could affect oscillations in other frequency bands; and (4) synchronization or desynchronization of specific endogenous oscillations by stimulating the electrodes, which could affect phase coherence in the stimulated regions (Antal and Herrmann, 2016). However, the optimal stimulation frequency is task-specific; beta-tACS improves short-term memory, gamma-tACS affects fluid intelligence, and theta-tACS improves WM, etc. (Santarnecchi et al., 2013; Jaušovec and Jaušovec, 2014; Feurra et al., 2016; Violante et al., 2017).

Working memory declines are distressing for the elderly and patients with neurological disorders. Although some pharmacological interventions might improve or prevent WM deficits in a particular group of patients, they can have serious side effects (Bogdanov and Schwabe, 2016). Thus, identifying techniques to prevent WM deficits that can be safely applied in humans is a pivotal and hot topic in neuroscience. Compared to rTMS, tACS demonstrates superior cost, tolerability, portability, and safety profile, making it an attractive potential tool for improving cognitive performance (Tavakoli and Yun, 2017; Bland and Sale, 2019). Although cognitive research with tACS is still in its infancy compared to rTMS, a number of studies have shown a promising WM enhancement effect, especially in the elderly and patients with cognitive deficits (Reinhart and Nguyen, 2019; Kehler et al., 2020; Chang et al., 2021). This review highlights the effect of tACS on WM, by presenting various methods and results of frequency-tuned tACS in healthy and unhealthy human adults, discussing established findings, unknowns, challenges, and perspectives important for developing stimulation protocols with promising safety and efficacy outcomes.

## Transcranial Alternating-Current Stimulation and Working Memory in Healthy Participants

### Theta-Transcranial Alternating-Current Stimulation

Among the models that have been addressed to understand the underlying mechanisms behind WM, one states that each gamma wave represents a single memory item (Sauseng et al., 2019). Therefore, the length of the theta cycle determines the number of gamma waves that can fit into a given theta cycle, and thus the WM capacity (Jensen and Lisman, 1996; Lisman and Jensen, 2013). It was found that the application of 4 Hz theta-tACS over the right parietal cortex improved the quality of memory representations, while 7 Hz theta-tACS impaired the quantity of WM (Guo et al., 2021). It has been demonstrated that theta cycles with low frequencies have a greater capacity to encode more gamma waves (Tseng et al., 2018). Wolinski et al. (2018) tested this hypothesis by slowing down and speeding up parietal theta frequencies by tACS to 4 and 7 Hz, respectively. They found that 4Hz theta-tACS enhanced WM capacity, whereas 7Hz theta-tACS had an unfavorable effect (Wolinski et al., 2018). The same findings were obtained by Jones et al. where theta-tACS of 4.5 Hz applied over fronto-parietal area improved WM outcomes better than 7 Hz (Jones et al., 2019). In addition, Vosskuhl et al. (2015) stimulated a wide network of the fronto-parietal cortices with below individual theta frequencies-tACS to probe the effect of such stimulation on memory performance. This protocol enhanced the capacity of STM but not that of WM (Vosskuhl et al., 2015). Bender et al. (2019) used the same experimental setup up utilized by Wolinski et al. (2018) but instead, the reference electrodes were positioned over Oz, Cz, and T8 (equidistant from the stimulation electrode P4), in this way, high-focal stimulation of the right posterior parietal cortex was achieved. Bender et al. (2019) aimed to achieve high-focal tACS over the parietal lobe and prevent the possible co-stimulation of the prefrontal or subcortical areas directly caused by the reference electrodes placed at frontal (Vosskuhl et al., 2015) or supraorbital site (Wolinski et al., 2018), respectively. The results of Bender et al. (2019) study are consistent with the results of the studies mentioned above that used different reference electrode montages (Wolinski et al., 2018; Jones et al., 2019).

Theta oscillations play an essential role in local processing and interregional functional connectivity, particularly in fronto-parietal regions (Roux and Uhlhaas, 2014; Fröhlich et al., 2015). Therefore, the cognitive effect of manipulating theta oscillations by the means of tACS has been extensively studied (Jaušovec and Jaušovec, 2014; Jaušovec et al., 2014; Meiron and Lavidor, 2014; Gonzalez-Perez et al., 2019; Abellaneda-Pérez et al., 2020; Sahu and Tseng, 2021). Two studies looking at the effect of theta-tACS on WM outcomes showed that theta-tACS administered to the right or left parietal cortex improved WM performance during backward recall task, while theta-tACS administered over left parietal cortex improved WM outcomes during visual-array comparison task (Jaušovec and Jaušovec, 2014; Jaušovec et al., 2014). The positive effect of online theta-tACS on WM performance was reported by Meiron and Lavidor (2014) who administered theta-tACS over right and left prefrontal cortices during verbal WM task. Sahu and Tseng (2021) pointed out that the WM enhancement effect of theta-tACS applied over the right fronto-parietal network was more pronounced in low-performers. Contrary to expectations, Chander et al. (2016) found that frontal midline theta tACS impaired WM outcomes in 2-back task, while Gonzalez-Perez et al. (2019) showed no cognitive enhancement effect of theta tACS administered over the occipital cortex for the perception and memory of facial and object stimuli. On the other hand, Röhner et al. (2018) compared the effect of tDCS, tACS, and sham stimulation administered over the fronto-parietal cortex in three separate sessions on the results of WM (2-back task) using a within-subject crossover design, where tasks were performed before, during, and after stimulation. The results of the study showed no differences in WM outcomes (reaction times and accuracy) according to the stimulation type. In an attempt to eliminate the practice effect (resulting from task repetition) on task results. A subgroup analysis was conducted with 10 participants who had received sham stimulation in the first session and showed similar cognitive performance on tasks administered before session 2 and session 3 (before tDCS and tACS). The results of the subgroup analysis indicated a cognitive improvement effect in favor of tACS stimulation (Röhner et al., 2018). The details of the studies are summarized in Supplementary Table 1.

Synchronization between brain oscillations of different brain cortices is considered an essential step in cognitive and behavioral processes (Klimesch et al., 2010; Siegel et al., 2012; Kleinert et al., 2017). Therefore, the effect of manipulating the phase synchrony of theta oscillations between cerebral cortices known to be involved in WM processes has been extensively addressed in the literature (Polanía et al., 2012; Alekseichuk et al., 2017; Kleinert et al., 2017; Violante et al., 2017; Tseng et al., 2018), the results of the studies can be outlined as follows: in-phase theta tACS over right and left posterior parietal cortices enhanced visual WM task scores in low-performers, while anti-phase theta-tACS had a detrimental effect on high-performers’ memory abilities (Tseng et al., 2018); Polanía et al. (2012) suggested that, in contrary to the anti-phase setting, in-phase theta tACS (between the left prefrontal and posterior parietal cortex) significantly reduced reaction times in a delayed letter discrimination task; Alekseichuk et al. (2017) induced fronto-parietal intra-hemispheric desynchronization and synchronization (theta-phase) and assessed the outcomes of 2-back visuospatial WM. They found that theta phase desynchronization had a negative impact on WM outcomes (reaction time and performance) and on phase connectivity (frontal and parietal cortices), but the synchronization of theta phases between the areas of interest had no effect on WM outcomes or electroencephalography (EEG) features (Alekseichuk et al., 2017); whereas Violante et al. (2017), examined the effect of in-phase and anti-phase theta-tACS on WM in different brain cortices (right fronto-parietal middle frontal gyrus and inferior parietal cortices) and suggested that in-phase theta-tACS improved n-back task outcomes when the cognitive demands were significantly high; in contrast to the results of the above studies, Kleinert et al. (2017) found no significant effects of theta-tACS (in-phase and anti-phase) applied over right fronto-temporal regions on visuospatial WM task outcomes or EEG features. On the other hand, Reinhart and Nguyen (2019) stimulated two brain regions by the mean of theta-tACS in healthy older adults using different experimental setups. They found that frontotemporal, in-phase theta-tuned (tuned to individual brain network dynamics)-tACS enhanced local theta/gamma PAC and WM task accuracy. Interestingly, the WM enhancement effect persisted for at least 50 min after stimulation (Reinhart and Nguyen, 2019). The studies are listed in Supplementary Table 1.

### Alpha, Beta, Gamma Transcranial Alternating-Current Stimulation

Gamma oscillations play an essential role in the maintenance and processing of WM (Honkanen et al., 2015). Therefore, the effect of gamma-tACS on WM outcomes has been extensively tested in healthy participants (Hoy et al., 2015; Tseng et al., 2016; Möller et al., 2017; Pahor and Jaušovec, 2018; Misselhorn et al., 2020). The results of the studies can be summarized as follows: gamma-tACS delivered over DLPFC selectively improved participants’ performance in tasks with higher cognitive load (3-back task), as suggested by Hoy et al. (2015). In three studies comparing the effect of theta-tACS and gamma-tACS on WM outcomes, it was found that neither theta-tACS nor gamma-tACS delivered over the left middle frontal gyrus (Santarnecchi et al., 2016), fronto-parietal cortex (Pahor and Jaušovec, 2018), or occipital cortex (Gonzalez-Perez et al., 2019) had reliable cognitive enhancement effect on WM outcomes. On the other hand, Tseng et al. (2016) suggested that unlike theta-tACS, left temporo-parietal anti-phase gamma-tACS boosted visual WM-binding outcomes in low-performers. Thompson et al. (2021) compared the effect of parietal gamma-tACS and parietal alpha-tACS on the outcomes of probabilistic retrospective cues task, with results indicating a cognitive enhancement effect in favor of gamma-tACS. Contrary to expectation, parietal gamma-tACS together with cognitive training (participants performed WM training with stimulation ∼25 min/day for five consecutive days) significantly impaired WM training-related gains (Möller et al., 2017). The details of the studies are summarized in Supplementary Table 1.

Alpha and beta oscillations are involved in different cognitive domains. Alpha oscillations are involved in the functional inhibition of visual processing areas, silencing task-irrelevant information and consequently improving the focus on task-relevant information processing (Klimesch et al., 2007; Bonnefond and Jensen, 2012; Popov et al., 2018). Due to the inhibitory function of alpha and beta oscillations, entrainment of these frequency bands would be beneficial for WM (Sauseng et al., 2009; Borghini et al., 2018). However, desynchronization of alpha and beta bands in the information processing regions while performing WM tasks could improve WM outcomes (Hanslmayr et al., 2019). Alpha-tACS administered over the parietal cortex improved target responses in retro-cue tasks in older participants (Borghini et al., 2018), whereas parietal alpha-tACS had no cognitive enhancement effect in young adults (Thompson et al., 2021). In a study that investigated the effect of beta, alpha, theta, and gamma tACS in young and middle-aged adults on forward memory span for digits, it was found that beta-tACS delivered over left posterior parietal cortex enhanced forward memory span for digits in young adults only (Feurra et al., 2016). Moreover, Misselhorn et al. (2020) examined the effect of beta-tACS and gamma-tACS administered over parietal cortex on delayed match-to-sample task outcomes in young and older adults, they found that gamma-tACS delayed the responses during the task, while beta-tACS speeded up the responses and that the effect was more pronounced in older adults. The details of the studies are summarized in Supplementary Table 1.

### Cross Frequency Coupling Transcranial Alternating-Current Stimulation and Working Memory

The coupling between theta phase and gamma amplitude has been hypothesized as a mechanism underpinning the WM process (theta/gamma neural code) (Lisman and Jensen, 2013; Roux and Uhlhaas, 2014). The CFC-tACS (cross-frequency coupling-transcranial-alternating current stimulation) protocols have been investigated in several cognitive domains (e.g., WM, learning, long term memory) (Lara et al., 2018; Turi et al., 2020; Riddle et al., 2021). Turi et al. (2020) found that theta/gamma trough-coupled tACS in the frontal cortex impaired cognitive performance in a Go/NoGo instrumental learning task based on monetary reward and punishment. On the other hand, Riddle et al. (2021) showed that delta/beta peak-coupled tACS and theta/gamma peak-coupled tACS in prefrontal cortex modulated cognitive task outcomes. Lara et al. (2018) examined the effect of theta/gamma CFC-tACS during encoding of long-term verbal memory. The results of this study were consistent with Turi et al. (2020) results (Lara et al., 2018). With regards to WM, Alekseichuk et al. (2016) investigated the effect of theta/gamma cross-frequency tACS, which was adapted to the intrinsic continuous theta and repetitive gamma waves in the prefrontal cortex, on the WM performance. The results of the study showed that theta/gamma tACS had a greater benefit on WM performance than theta tACS alone. Gamma bursts above theta peaks significantly improved WM compared to gamma bursts above theta troughs, and the gamma frequencies associated with the optimal results were in the range of 80–100 Hz (Alekseichuk et al., 2016).

## Transcranial Alternating-Current Stimulation and Working Memory in Patient Population

Transcranial alternating-current stimulation modulates cognitive processes in the brain by entraining endogenous oscillations and inducing long-lasting synaptic plasticity (Markram et al., 1997). tACS has only recently been introduced into psychiatric clinical trials and studied for its potential therapeutic role in the treatment of patients with schizophrenia (Hoy et al., 2016; Kallel et al., 2016; Sreeraj et al., 2017, 2019, 2020; Mellin et al., 2018; Ahn et al., 2019; Force et al., 2021), depression (Alexander et al., 2019; Wilkening et al., 2019; Riddle et al., 2020a), obsessive-compulsive disorder (Klimke et al., 2016), ADHD (Dallmer-Zerbe et al., 2020), Alzheimer’s disease (Benussi et al., 2021), and dementia (Naro et al., 2016; Moussavi et al., 2021). Abnormal gamma activity has been observed in various neuropsychiatric disorders (Herrmann and Demiralp, 2005; Uhlhaas and Singer, 2006). Therefore, the gamma-tACS effect on WM outcomes has been examined in patients with these disorders (Sreeraj et al., 2017; Haller et al., 2020b,a; Kehler et al., 2020; Kim et al., 2021). Specifically, the effect of tACS on WM outcomes in patients with neurological disorders has been investigated in the following disorders: schizophrenia 6 studies, summarized as follows: in-phase theta-tACS administered over fronto-parietal cortex (Sreeraj et al., 2017, 2019; Chang et al., 2021) improved WM outcomes and the effect was more sustained (50 days) when theta-tACS was administered daily (20 min/day) for 5 days. Contrary to expectations, no enhancement effect on memory was observed in two studies when single-session gamma-tACS was applied over the DLPFC (Hoy et al., 2016; Papazova et al., 2020), or when in-phase gamma-tACS was administered over the fronto-parietal cortex (Sreeraj et al., 2017). Whereas multiple sessions of gamma-tACS administered over several days produced positive cognitive results (Haller et al., 2020a). In patients with cognitive impairment and dementia (four studies), all studies investigating the effect of gamma-tACS delivered over the DLPFC or left angular gyrus showed an improvement in cognitive outcome from either single or multiple stimulation sessions, and the effect persisted for 1 month when multiple gamma-tACS sessions were applied over the DLPFC (30 min/day–5 days/week for 4 weeks) and combined with cognitive exercises (Kehler et al., 2020; Bréchet et al., 2021; Kim et al., 2021; Moussavi et al., 2021). In addition, the application of gamma-tACS over the prefrontal cortex for 10 days improved WM in patients with MDD (1 study) (Haller et al., 2020b). In a study conducted to evaluate the effect of tACS at 35 and 90 Hz on the visual and pitch memory outcomes in patients with congenital amusia, 35 Hz-tACS was found to selectively improve pitch memory in patients with congenital amusia (Schaal et al., 2015). The details of the studies are summarized in Table 2.

**TABLE 2:** Summaries of the studies investigating the effect of frequency-tuned transcranial alternating-current stimulation on cognitive outcomes in unhealthy participants.

Study	Description	Electrode(s) location(s)	Task(s)	Main outcome(s)
**Schizophrenia**				
Chang et al., 2021	Thirty-six patients with schizophrenia Theta-tACS (6 Hz) Two groups (sham and verum) Twice daily, 6 Hz-tACS-20 min sessions of in-phase fronto-parietal tACS or sham stimulation for five consecutive weekdays. WM task during stimulation Follow-up for 1 month	Frontal and parietal cortices	Positive and Negative Syndrome Scale 2-back task	Theta-tACS improved the negative symptoms of schizophrenia for at least one month post intervention Theta-tACS enhanced WM capacity.
Sreeraj et al., 2017	Case report One patient with schizophrenia Two sessions 2 days apart Session 1 (online 6Hz-tACS) in-phase Session 2 (online 40 Hz-tACS) in-phase	Left DLFPC and left posterior parietal region F3 and P3	Sternberg’s task	6 Hz-tACS boosted WM performance
Sreeraj et al., 2019	Case report One patient with schizophrenia Five once-daily tACS (20 min/day) In-phase 6Hz -tACS	Left DLFPC and left posterior parietal region F3 and P3 respectively	Cognitive tasks (WM and attention)	Cognitive enhancement effect in WM and other cognitive domains Long-lasting aftereffect for 50 days
Hoy et al., 2016	Ten patients with schizophrenia Three sessions 3 days apart 40 Hz-tACS, sham, and tDCS	Left DLPFC	Two-back WM task	Contrary to the expectations, no enhancement effect on memory was observed by using gamma-tACS Performance was significantly enhanced by gamma-tDCS and sham stimulations
Haller et al., 2020a	Three patients with schizophrenia Twice-daily 10 min prefrontal gamma-tACS stimulation for 10 days Assessment at baseline, after five days of stimulation and after the last session of stimulation	DLPFC	Trail-making test and word fluency Positive and Negative Syndrome Scale	Gamma-tACS improved the Positive and Negative Syndrome Scale Prefrontal gamma-tACS improved trail-making test performance and word fluency
Papazova et al., 2020	Fifteen patients with schizophrenia— Gamma -tACS and sham stimulation	DLPFC F3 and F4	Verbal n-back task (1- to 3-back conditions)	No significant effect of gamma-tACS in terms of d prime values and reaction times. An inverse association between cognitive load and reaction time was detected during gamma-tACS as compared to sham stimulation
**Mild cognitive impairment and dementia**				
Moussavi et al., 2021	Twenty-eight older adults with dementia Two groups (cognitive exercise alone -group 1-, cognitive exercise + 40 Hz-tACS- group 2- for 4 consecutive weeks, (5 days/week, two 30 min-sessions/day) -Assessment at bassline, post-intervention and one month after the end of the intervention (follow up)	Left DLPFC	independent assessment (WMS-IV) as the primary outcome measure	Evaluation after intervention showed cognitive improvement in both groups. Cognitive exercise + 40 Hz-tACS resulted in better cognitive improvement one month post intervention compared to group 1
Kehler et al., 2020	Seventeen older adults with mild to moderate dementia or mild cognitive impairment Eleven participants received 40Hz -tACS + cognitive exercises (30 min/day–5 days/week for 4 weeks) (tACS group) Six participants received only cognitive exercises (non-tACS group)	Left DLPFC	Wechsler Memory Scale (WMS-IV) post intervention and after 1 month	Improved cognitive functions were observed in both groups immediately after the intervention The improvement in cognitive functions was maintained in the tACS group for 1 month after the intervention, compared with the non-tACS group
Kim et al., 2021	Twenty MCI patients The study compared between tDCS and gamma-tACS for cognitive improvement in MCI patients Cognitive assessment (task) and EEG analysis were done before and after the single- session stimulation	DLPFC	Stroop and Trail-making-tests	Gamma-tACS -single session- over DLPFC improved the stroop-color compared to tDCS and sham stimulation Gamma-tACS-single session- enhanced trait- making test compared to sham stimulation EEG analysis showed enhancement of beta activity and increased beta 2 source activity in the anterior cingulate after gamma-tACS-tDCS decreased delta and theta activity compared to sham stimulation
Bréchet et al., 2021	Two patients with Alzheimer’s disease-related dementia Patient-tailored home-based tACS delivered daily to patients and remotely monitored by the study team Multi-week regimen of daily sessions of 40 Hz tACS to the left angular gyrus Caregivers delivered tACS (tACS administrator) 20 min tACS/day- 5 days per week, for 14 weeks (70 sessions in total)	Left angular gyrus	Baseline dementia severity was assessed using the Clinical Dementia Rating scale (36) The Montreal Cognitive Assessment (MoCA)	During the 14 weeks of the study, both participants showed improvement from baseline in the testing administered every 2 weeks
**Depression**				
Haller et al., 2020b	Six patients with major -depression 40 Hz-tACS Two groups (group 1: two 10-min stimulations (separated by a 3 h interval) and group 2 20-min stimulation Bothoth groups received daily on weekdays for 2 weeks (total 10 day) Objective and subjective assessment at baseline, at day 5 of stimulation, at the last day of stimulation	Left and right prefrontal cortex	Hamilton Depression Rating Scale and Beck Depression Inventory Three-back task and word fluency	40 Hz-tACS over prefrontal cortex for 10 days improved the cognitive functions assessed by word fluency and n-back test in both groups Hamilton Depression Rating Scale and Beck Depression Inventory decreased during the stimulation in both groups
**Congenital amusia**				
Schaal et al., 2015	Online gamma-tACS Nine patients with congenital amusia and 8 matched controls Two tACS sessions (35Hz or 90Hz) Completed tasks before and during stimulation	Right DLPFC	Pitch-memory task Visual WM task	35 Hz-tACS over the right dorsolateral prefrontal cortex significantly enhanced pitch memory performance (accuracy) in patients with congenital amusia No enhancement effect on visual WM and for 90Hz stimulation

*tACS, transcranial alternating-current stimulation; Hz, hertz; WM, working memory; DLPFC, dorsolateral prefrontal cortex; MCI, mild cognitive impairment; EEG, electroencephalography; tDCS, transcranial direct-current stimulation.*

Interestingly, Bréchet et al. (2021) investigated the feasibility, efficacy, and safety of patient-tailored, home-based gamma-tACS delivered over the left angular gyrus and administered over 14 weeks (70 sessions in total) in two patients with Alzheimer’s disease-related dementia. In this study tACS sessions were delivered by trained caregivers and the administration can be remotely monitored by the study team. Results from this study suggest that both participants’ cognitive abilities improved from baseline on tests administered every 2 weeks during the 14 weeks of the study and that the overall protocol is safe and feasible. The results of the study provide an incentive for future randomized-controlled trials with large sample sizes to investigate the effect of tACS in a real-world setting (Bréchet et al., 2021). The details of the study are summarized in Table 2.

## Discussion

### Frequency-Tuned Non-invasive Brain Stimulation and Working Memory

Non-invasive brain stimulation, particularly frequency-tuned stimulation, is a recent approach that relies on the entrainment of endogenous oscillations to causally alter cognition and behavior. According to the dynamic systems theory, entrainment of the endogenous oscillations is strongest when the frequencies of the entraining stimulus match the ongoing brain frequencies (Ali et al., 2013; Vossen et al., 2015). The resonance phenomenon implies that matching the ongoing brain oscillations responsible for a given cognitive task with the frequency of the stimulus leads to an increase in the activity and coherence of these networks-neuronal synchronization — (Herman et al., 2013), which in turn leads to behavioral changes in activities supported by these neuronal networks (Borghini et al., 2018). rTMS and tACS have been used to modulate ongoing oscillation in a frequency-specific manner. In this review, we have focused on the effect of tACS on WM outcomes as it is less expensive, more tolerable, more portable, and safer compared to rTMS.

Cognitive deficits are evident in elderly and patients with schizophrenia, ADHD, MDD, MCI, and Alzheimer’s disease. Patients with dementia and Alzheimer’s disease are thought to have functional impairments in the central executive component of the WM model (Baddeley et al., 1991). Older adults are unable to optimally ignore task-irrelevant information, which is considered a fundamental step in WM processing (Borghini et al., 2018). Slowing, preventing, and reversing cognitive decline and dementia are challenging (Yao et al., 2020). Recent studies have tested the effects of lifestyle changes, cognitive training, exercise, and diet on cognitive functions, the results of the studies are mixed. Although a positive effect was found, large, well-conducted studies are needed to draw a conclusion (Bhatti et al., 2020). NIBS techniques induce long-lasting changes in cortical excitability by enhancing synaptic plasticity (Terranova et al., 2019). Responses to NIBS may vary depending on several factors, such as exercise, as regular aerobic exercise can modify plasticity and improve the response to NIBS (Kramer and Erickson, 2007). Aging alters the capacity of processes important for synaptic plasticity, so that NIBS-induced plasticity decreases with age in both healthy and neurologically impaired individuals (Müller-Dahlhaus et al., 2008). Furthermore, NIBS-induced plasticity can be significantly modulated by many neuropharmacological agents (Möhler, 2006; Ridding and Ziemann, 2010). Due to differences in brain dynamics and cortical activities, healthy participants and patients with neurological disorders respond differently to NIBS. Moreover, impaired long-term plasticity in patients with chronic diseases lead them to respond differently to NIBS aftereffects compared to patients with acute illnesses (Uhlhaas and Singer, 2012; Hoy et al., 2015, 2016; Dallmer-Zerbe et al., 2020).

### Transcranial Alternating-Current Stimulation and Working Memory

The effect of frequency-tuned tACS on WM outcomes in healthy participants has been addressed in many studies (Santarnecchi et al., 2016; Tseng et al., 2016; Möller et al., 2017; Pahor and Jaušovec, 2018), but a limited number of studies investigated the cognitive effects of tACS in the elderly, patients with neuropsychiatric and neurodegenerative disorders (Hoy et al., 2016; Kehler et al., 2020; Papazova et al., 2020). The effects of frequency-tuned tACS on WM are inconsistent, especially when tACS is applied to young, healthy participants or in a single-session (Hoy et al., 2015; Sreeraj et al., 2017; Papazova et al., 2020). Such a substantial diversity in results could be due to the inter-and intra-individual variability as well as differences in the experimental setups. Inter-individual variability could be partly due to individual differences in the theta/gamma ratio, theta and gamma frequencies, and the shape of the theta peak (sharp or broad), and participants’ baseline characteristics (e.g., low-performer versus high-performer to cognitive task administered prior to stimulation), elderly versus young, healthy versus unhealthy, etc. On the other flip, variability in task type and cognitive load explains intra-individual differences in sensitivity to the tACS effect (Wolinski et al., 2018). In addition, the relative release of sex hormones at different phases of the menstrual cycle has been suggested to influence women’s cognitive responses (Berman et al., 1997; Amin et al., 2006). Therefore, in two studies conducted by Jaušovec and Jaušovec (2014) and Jaušovec et al. (2014) the subjects participated in two sessions—a sham session and a tACS session. The sham and verum sessions were 28 days apart (on the same day of the menstrual cycle) to minimize the confounding effect of hormones on the measured cognitive outcomes. In addition, the cortical excitability of females is similar to males only during follicular phase of the menstrual cycle (Inghilleri et al., 2004). Thus, Fertonani et al. (2011) and Gonzalez-Perez et al. (2019) examined the effect of tACS in females only during this phase. However, the cognitive effects of sex hormones need to be studied in-depth before they can be considered as a factor influencing clinical outcomes. Differences in the experimental setups such as the number and duration of tACS sessions, stimulation parameters (frequency, intensity, duration, etc.) and the specific sub-region of the cortex that is stimulated are factors that influence the tACS entrainment effect (Liu et al., 2017).

The WM enhancement effect of tACS is more pronounced when applied over the prefrontal or parietal cortex in the theta or gamma frequency range (Hoy et al., 2015; Tseng et al., 2016; Sreeraj et al., 2019; Kehler et al., 2020) and when applied to participants performing tasks with relatively high cognitive demands (e.g., the enhancement effect was more pronounced in the 3-back task than in the 2-back task) (Hoy et al., 2015; Papazova et al., 2020). In addition, the cognitive effect of tACS is more evident in low-performers such as the elderly and patients with neurodegenerative diseases, who benefited from tACS administered either in one or in multiple sessions (Borghini et al., 2018; Reinhart and Nguyen, 2019; Kehler et al., 2020; Misselhorn et al., 2020; Bréchet et al., 2021; Moussavi et al., 2021). On the other hand, patients with schizophrenia or MDD may require multiple tACS sessions administered over several days to achieve cognitive benefits (Sreeraj et al., 2019; Haller et al., 2020a,b; Chang et al., 2021). The long-term cognitive effect (e.g., the effect lasting one month after stimulation) of tACS could be achieved by multiple tACS sessions administered over several days or weeks (e.g., 20 min/day for five consecutive days/week for 4 weeks) (Sreeraj et al., 2019; Chang et al., 2021; Moussavi et al., 2021). However, most tACS studies that have targeted neurological patients are case reports and case series conducted with small numbers of participants (Sreeraj et al., 2017, 2019; Haller et al., 2020a,b). Interestingly, there are currently several ongoing randomized, double-blind studies investigating the long-term safety and efficacy of tACS in patients with mild Alzheimer’s disease (TRANSFORM-AD study) (Xing et al., 2020), depression after stroke (SPIRIT Compliant study) (Wang H. et al., 2020), and MDD (Wang H. X. et al., 2020; Huang et al., 2021). Follow-up in these studies will continue for up to several weeks after the end of stimulation (Wang H. et al., 2020; Wang H. X. et al., 2020; Xing et al., 2020; Huang et al., 2021). Among the tACS protocols that have recently gained popularity among researchers is the CFC-tACS. During cognitively demanding tasks, endogenous gamma bursts nest into theta peaks in the frontal cortex (Peak-coupled CFC) (Smith et al., 2015). Thus, manipulating this endogenous coupling between theta and gamma frequencies by the mean of tACS could affect WM outcomes. The causal role of CFC in cognitive processes has been investigated in several studies (Alekseichuk et al., 2016; Lara et al., 2018; Berger et al., 2019; Bramson et al., 2020; Hermiller et al., 2020; Turi et al., 2020). In the context of WM, the causal role of CFC was tested by Alekseichuk et al. (2016) by matching exogenously applied theta and gamma CFC-tACS to intrinsic continuous theta and repetitive gamma waves in the prefrontal cortices of healthy participants. They found that theta/gamma peak-coupled tACS enhanced WM more than theta-tACS alone, and the effect was more evident when gamma bursts (range of 80–100 Hz) were coincided into theta peaks (peak-coupled tACS) (Alekseichuk et al., 2016). The effect of CFC-tACS on cognitive functions varies depending on the brain area stimulated, the cognitive domain tested (WM, learning, long-term memory, etc.), the frequency bands targeted, and the experimental design used (Alekseichuk et al., 2016; Lara et al., 2018; Turi et al., 2020; Riddle et al., 2021). However, CFC-tACS needs further investigation in healthy individuals and patients with cognitive deficits due to its promising cognitive effects.

## Future Directions and Limitations

The current studies have a number of limitations, summarized as follows: (1) Most studies currently reporting the cognitive effects of tACS are case reports, case series, and pilot studies conducted on a small number of participants, indeed, randomized, placebo-controlled, and double-blind studies on a large number of volunteers are needed to elucidate the WM enhancement effect of tACS on WM outcomes. (2) Only one study conducted by Bréchet et al. (2021) examined the effect of home-based tACS on cognitive outcomes in two patients with dementia. Although the effect of tACS on WM performance is promising, translating tACS effect to the real-world is challenging. This is because real-world conditions are dynamic, complex, and generally noisier than in the laboratory. However, mobile EEG devices that allow EEG recordings outside the laboratory would be beneficial, besides behavioral assessments, to evaluate the effect of tACS in realistic situations in future studies (Debener et al., 2015; Bleichner and Debener, 2017). (3) Most studies did not use personalized stimulation parameters. The use of personalized tACS parameters such as stimulation intensities and frequencies would lead to more effective stimulation (Jaušovec and Jaušovec, 2014; Jaušovec et al., 2014; Vosskuhl et al., 2015; Reinhart and Nguyen, 2019) with minimal side effects (Thut et al., 2011b; Deng et al., 2013) and reduce the heterogeneity of results due to the inter-individual variability (section “tACS and Working Memory”). (4) There are a limited number of studies in the literature on the tACS effects on patients with neuropsychiatric disorders and cognitive deficits. (5) Lack of consistent aftereffect. Although multiple sessions of tACS delivered over several weeks may elicit long-lasting cognitive effects (e.g., at least 1-month post-stimulation), more and more studies are needed to replicate the results and determine the maximum duration of the cognitive effect.

Interestingly, all NIBS studies that targeted two regions in healthy older adults significantly improved WM outcomes (Park et al., 2014; Arciniega et al., 2018; Borghini et al., 2018; Reinhart and Nguyen, 2019), while those that targeted one region showed mixed results (Goldthorpe et al., 2020). Therefore, it is reasonable to call for studies targeting two regions, both in healthy participants and in patients with schizophrenia, depression, dementia, MCI, etc. using tACS.

In summary, recent studies suggest that participants with lower WM capacity due to advanced age or neurological disorders tend to derive greater cognitive benefit from tACS stimulation. These participants benefited from theta-tACS and/or gamma-tACS delivered over the prefrontal cortex. Schizophrenic patients may require multiple stimulation sessions to benefit from the cognitive effects of tACS. Elderly people and patients with neurodegenerative diseases, on the other hand, may benefit from one or more stimulation sessions. In addition, multiple tACS sessions may result in long-lasting cognitive improvement in participants with cognitive deficits. However, the current studies have been limited by small sample sizes, heterogeneous methods, inconsistent long-term effects, and so on. Further large sample sizes and well-designed studies are needed to confirm the cognitive effects of tACS and to determine optimal stimulation protocols for administration to patients with cognitive impairment. Comprehensive studies aimed at: (1) targeting more and more neuropsychiatric patients and patients with cognitive impairment; (2) achieving consistent aftereffects; (3) using personalized stimulation parameters; (4) translating the effects of tACS into realistic situations; (5) targeting two regions simultaneously (e.g., the frontal and parietal cortices); (6) investigating the effect of CFC-tACS on WM using different experimental setups are needed before frequency-tuned NIBS could alter cognitive impairments in everyday life.

## Author Contributions

WA conducted the literature search and the summaries of previous published studies. MA and WA wrote the first and the final version of the manuscript. EK contributed to the progress of the manuscript and provided guidance with expert perspective. All authors contributed and approved the final version of the manuscript.

## Conflict of Interest

The authors declare that the research was conducted in the absence of any commercial or financial relationships that could be construed as a potential conflict of interest.

## Publisher’s Note

All claims expressed in this article are solely those of the authors and do not necessarily represent those of their affiliated organizations, or those of the publisher, the editors and the reviewers. Any product that may be evaluated in this article, or claim that may be made by its manufacturer, is not guaranteed or endorsed by the publisher.

## Abbreviations

WM: working memory
STM: short-term memory
DLPFC: dorsolateral prefrontal cortex
ACC: anterior cingulate cortex
ADHD: attention-deficit/hyperactivity disorder
MDD: major depressive disorder
MCI: mild cognitive impairment
Hz: hertz
CFC: cross-frequency coupling
PAC: phase-amplitude coupling
NIBS: non-invasive brain stimulation
tES: transcranial electrical stimulation
TMS: transcranial magnetic stimulation
tDCS: transcranial direct current stimulation
tACS: transcranial alternating current stimulation
tRNS: transcranial random noise stimulation
rTMS: repetitive transcranial magnetic stimulation
TBS: theta burst stimulation
cTBS: continuous theta burst stimulation
iTBS: intermittent theta burst stimulation
FDA: Food and Drug Administration
STDP: spike-timing-dependent plasticity
EEG: electroencephalography
EPR: event related potential
MEG: magnetencephalography
fMRI: functional magnetic resonance imaging
CFC-tACS: cross-frequency coupling- transcranial alternating current stimulation.
