# Supplementary material for: Working Memory and Transcranial-Alternating Current Stimulation—State of the Art: Findings, Missing, and Challenges
Source: Front Psychol. 2022 Feb 14;13:822545. doi: 10.3389/fpsyg.2022.822545 (PMC8882605; doi:10.3389/fpsyg.2022.822545)
Supplement: Supplementary file 1 [file Table_1.docx]

**Supplementary Table 1**: Summaries of the studies investigating the effect of frequency-tuned transcranial alternating-current stimulation on working memory outcomes in healthy participants. tACS: transcranial alternating-current stimulation; Hz: Hertz; WM: working memory; EEG: electroencephalography; STM: short term memory; EPR: event related potential; DLPFC: dorsolateral prefrontal cortex; tDCS: transcranial direct-current stimulation; MEG: magnetencephalography; fMRI: functional magnetic resonance imaging; HD-tACS: high-definition- transcranial alternating-current stimulation

| **Study** | **Description** | **Electrode(s) location(s)** | **Task(s)** | **Main outcome(s)** |
| --- | --- | --- | --- | --- |
| **Theta-tACS** | | | | |
| (Wolinski et al., 2018) | - Theta-tACS - 16 participants - Three stimulation sets: 4Hz theta -tACS,7Hz-theta tACS, and sham stimulation | - Right parietal cortex - Return electrode: - Control montage: over the vertex - Experimental montage: over the right supraorbital region | - Visual delayed match-to sample task | - 4Hz tACS improved WM capacity - 7Hz -tACS reduced WM capacity |
| (Jones et al., 2019) | - 68 healthy participants - 2 experiments - Exp.1: theta (fast) 7 Hz-tACS and alpha 11 Hz-tACS - Exp.2: theta (slow) 4.5 Hz-tACS | - Experiment: right fronto-parietal - Experiment: fronto-parietal or bilateral montages | - 3-back WM task (object and spatial) | - Exp.1: no behavioural enhancement effect - Exp.2: enhancement in object WM with right fronto-parietal 4.5 Hz (slow) theta-tACS. |
| (Vosskuhl et al., 2015) | - Below individual theta-tACS - 33 healthy participants - Two groups (sham versus verum stimulation) - EEG was recorded before and after the stimulation - STM and WM capacities were obtained before, during and after the stimulation - Below individual theta-tACS | - Pz and FCz - Return electrode: central electrode | - Digit span task (backward and forward) - 3-back task | - Below individual theta-tACS enhanced STM capacity in forward digit span task - No enhancement effect of theta-tACS on WM performance using - backward digit span task or 3-back task |
| (Bender et al., 2019) | - 32 participants - Received theta-tACS at 4Hz,7Hz and sham stimulation | - Right parietal cortex - Return electrodes: Oz, Cz and T8. | - Visuospatial delayed match-to-sample task | - Increased WM capacity was detected for visual field contralateral to the stimulation with 4Hz tACS. |
| (Jaušovec and Jaušovec, 2014) | - Individually determined theta-tACS - 24 subjects divided into 2 groups: parietal and frontal. - The 2 groups were equalized in terms of sex and performance - Sham and verum stimulations separated by 28 days - ERP component analysis | - Left parietal and left frontal cortices - Return electrode: - above the right eyebrow | - Visual-array comparison task | - Left parietal theta-tACS significantly increased WM storage capacity as compared to sham stimulation - ERP P300 latency decrease was associated with increased WM capacity. |
| (Jaušovec et al., 2014) | - Individual determined theta -tACS - 36 volunteers divided into 3 groups -left parietal, right parietal and left frontal - The 3 groups were equalized in terms of sex and performance - Sham and verum stimulations separated by 28 days. | - Left frontal, left parietal, and right parietal cortices (offline) - Return electrode: above the right eyebrow | - Forward and backward Corsi block-tapping tasks (spatial WM) - Forward and backward digit-span tasks (verbal WM) - n-back tasks | - Theta-tACS over right or left parietal cortex enhanced WM capacity. - Left parietal theta-tACS had a more pronounced effect on backward recall (not related to task content (spatial or verbal)) as compared to forward recall tasks - Right parietal theta-tACS had a greater effect on n-back performance particularly (2 back condition). |
| (Meiron and Lavidor, 2014) | - Theta-tACS - 24 female participants - Two groups (sham and active stimulation groups) | - Left and right DLPFC | - Verbal WM task | - Theta -tACS had a positive effect on WM accuracy in comparison to sham stimulation. |
| (Sahu and Tseng, 2021) | - 20 healthy participants - Theta-tACS to the right fronto-parietal cortex - Reto-cuing with theta-tACS to investigate whether tACS effect is stage-specific | - Right fronto-parietal | - Stage-specific change detection paradigm | - The enhanced effect of theta-tACS was more pronounced in low-performers - Theta-tACS enhanced visual WM outcomes in a non-stage dependent manner |
| (Chander et al., 2016) | - 20 volunteers - Volunteers were divided into 2 groups (A and B groups) - Group A (Individualized frontal midline theta-tACS over prefrontal cortex) - Group B (sham stimulation) - MEG study | - Frontal midline region (medial prefrontal and medial parietal areas (FPz and Pz)) | - 2-back task | - -Individualized frontal midline theta-tACS reduced WM performance and theta activity in the midline region - -Theta-tACS block midline theta power increase in response to WM demands. |
| (Röhner et al., 2018) | - Theta-tACS, tDCS, and sham stimulation in a counterbalanced order - within-subject, crossover design was applied - 30 healthy adults - tACS to the fronto-parietal region and tDCS to the DLPFC - WM task was performed before, during and after the stimulation | - Frontal and parietal cortices | - Visual 2-back WM task | - No differences in reaction times and accuracy were observed according to the stimulation type. - A marked practice observed from repeating the task before, during, and after the stimulation - Subgroup analysis for 10 participants (who received sham stimulation in the first session) and had the same cognitive performance before session 2 and session 3 (tACS and tDCS sessions) - The subgroup analysis showed an enhancement effect in favour   of tACS stimulation |
| (Tseng et al., 2018) | - In-phase and anti-phase theta (6 Hz) tACS - 48 participants - 2 groups (in-phase and anti-phase (180-degree difference between right and left parietal electrodes). - Sham and verum stimulations were held in two days separated by at least one week | - Left and right posterior parietal cortices - Reference electrode: lower left cheek | - Visual WM task | - In-phase theta tACS enhanced only low-performers` outcome (visual WM performance) - Theta anti-phase stimulation had a detrimental effect on high- performers’ memory abilities and no significant effect on low- performers. |
| (Polanía et al., 2012) | - Theta–tACS - Investigated left fronto-parietal long-range functional connectivity and its effect on cognitive performance - Verum and sham stimulations - EEG and ERP analysis. | - Left DLPFC - and left posterior parietal cortices - P3 and F3 - Reference electrode: Cz | - Delayed letter-discrimination task | - In-phase theta-tACS significantly reduced reaction times at the memory matching period - Anti-phase theta-tACS had a detrimental effect on performance. |
| (Alekseichuk et al., 2017) | - Theta-tACS - 35 participants - Natural history group and experimental group - Experimental group: divided into 3 groups: synchronization group, desynchronization, and sham stimulations. | - Right and left fronto-parietal cortices - Reference electrode: over the right earlobe | - 2-back visuospatial task | - Fronto-parietal intra-hemispheric desynchronization caused a decrease in WM performance and an increase in reaction times - Decreased phase connectivity between frontal and parietal cortices induced by theta-tACS desynchronization. - Fronto-parietal theta-tACS synchronization induced no changes in EEG or behavioural outcomes. |
| (Violante et al., 2017) | - 34 participants - In-phase and anti-phase theta-tACS and sham stimulations - fMRI | - Right fronto-parietal - (middle frontal gyrus and inferior parietal areas F4 and P4 electrodes) - Return electrode: T8 (middle temporal gyrus) | - Verbal working memory - Choice reaction task - N-back task | - In-phase theta-tACS improved WM performance when the cognitive demands were significantly high (decreased reaction time for 2-back but not 1-back tasks). - In-phase theta-tACS during the verbal WM tasks increased parietal activities. |
| (Kleinert et al., 2017) | - 18 participants - In-phase, anti-phase and sham tACS stimulation (frequency 5 Hz) - EEG analysis. | - Right fronto-temporal (F4 and P4) - Return electrode: Cz | - Visuospatial match-to-sample task | - No significant effects of tACS on performance using different (in-phase and anti-phase) protocols on behavioural and EEG outcomes. |
| (Reinhart and Nguyen, 2019) | - Older adults aged 60–76 years. - Theta-tACS (2 sites) in phase - Younger adults: participated in sham stimulation only - Older adults: participated in sham and active stimulation - tACS stimulation while the older adults performed 10 blocks of a change detection task (the task consisted of 30 blocks) - Fronto-temporal in-phase theta tACS (tuned to the individual brain network dynamics)- tuned tACS - 4 experiments were conducted under different conditions: sham stimulation, fronto-temporal in-phase theta-tuned stimulation, unifocal frontal theta-tuned stimulation, unifocal temporal theta-tuned stimulation - Fronto-temporal in-phase 8Hz non-tuned stimulation, fronto-temporal anti-phase theta-tuned HD-tACS. | - Left prefrontal and left temporal cortices | - Change-detection task - 10 blocks during stimulation and then the participants completed 20 blocks post stimulation | - Fronto-temporal in-phase theta-tuned -tACS enhanced local theta/gamma coupling in older adult. - Fronto-temporal in-phase theta-tuned-tACS enhanced WM task accuracy. - Frontotemporal in-phase theta-tuned-stimulation enhanced theta-phase synchronization in the tested group. - The WM improvement persisted for at least 50 minutes after 25 minutes of theta stimulation. |
| **Alpha, beta and gamma tACS** | | | | |
| (Hoy et al., 2015) | - Offline gamma-tACS - tDCS, and sham stimulation - 18 participants - WM performance was tested prior to and post the stimulation - Concurrent with stimulation participants underwent two 5 min blocks of the 2-back WM task concurrently with the stimulation (Intra-stimulation task). | - Left dorsolateral prefrontal cortex F3 - Reference electrode: contralateral supraorbital area | - 2- and 3-back task | - As compared to other protocols, gamma-tACS selectively improved participants’ performance in 3-back tasks |
| (Santarnecchi et al., 2016) | - 58 participants - 2 groups - Group1: gamma, theta, and sham -tACS during WM and fluid intelligence tasks - Group2: same protocols as in group 1 but theta stimulation was replaced by high frequency transcranial random white noise stimulation (101-640Hz). | - Left middle frontal gyrus - Return electrode: Cz | - Fluid intelligence task - Working memory task | - No enhancement effect of on performance (reaction time and accuracy) were detected during WM tasks - Gamma-tACS reduced reaction times during fluid intelligence task. |
| (Pahor and Jaušovec, 2018) | - Theta-tACS, gamma-tACS and sham stimulation - Each stimulation was performed on separate day. - 72 healthy female participants - Pre- and post-stimulation resting EEG recording. - Performance of behavioural tasks during EEG recording. - ERP analysis | - Bilateral frontal, bilateral parietal, left fronto-parietal and right fronto-parietal - 4 groups; group 1 (P3–P4), group 2 (F3–P3), group 3 (F4–P4), and group 4 (F3–F4). - Leads were referenced - to linked mastoids (A1 and A2) - Ground electrode: forehead | - 2 change detection tasks (figural and verbal) - four n-back tasks (figural and verbal) 2 and 3-back conditions | - No significant effect of gamma-tACS on WM performance - Inconsistent effect of theta-tACS on WM outcomes of n-back tasks - Theta-tACS over bilateral parietal and right fronto-parietal cortices significantly affected P3 amplitude and latency during n-back tasks. - Theta amplitude was affected by theta-tACS. |
| (Gonzalez-Perez et al., 2019) | - 60 healthy adults - Gamma-tACS and theta-tACS - Sham and active stimulation - The effect of gamma-tACS on the face and object perception and memory outcomes | - Occipital cortex | - Face perception task - Object perception task - Cambridge face memory task -Cambridge car memory task | - Gamma-tACS over the left frontal-right occipital enhanced faces and objects perceptions but not memory      - No enhancement effect (perception or memory) was observed using theta-tACS |
| (Tseng et al., 2016) | - Theta and gamma tACS - 20 participants - Binding visual WM performance measurement - Exp.1: anti-phase gamma tACS over left temporoparietal regions. - Exp.2: in-phase gamma tACS over left temporoparietal regions. - Control exp.: in-phase and anti-phase theta-tACS over left temporoparietal regions. | - Left temporal and parietal cortices - Reference electrode: right cheek | - Visual WM task | - Left temporoparietal anti-phase gamma-tACS boosted visual WM-binding outcomes in low-performers |
| (Thompson et al., 2021) | - 51 young adult participants - Gamma 35 Hz-tACS, versus alpha 10 Hz-tACS versus sham stimulation - Tested memory-based attention and inhibitory abilities. | - Bilateral parietal | - Probabilistic retrospective cues | - Parietal gamma-tACS enhanced working memory recall precision significantly compared to alpha-tACS and sham stimulations - Post hoc analysis showed that gamma-tACS improved memory precision only in high-baseline performers |
| (Möller et al., 2017) | - 60 healthy young adults - 5 days of real or sham stimulation - tDCS versus gamma-tACS over bilateral frontal or parietal cortices - 5 days of visuo-spatial WM training and stimulation - participants preformed WM training for 5 consecutive days for about 25 min while receiving stimulation | - Bilateral frontal or parietal lobe | - WM task - number of remembered spatial position). | - WM impairment associated with parietal tACS and frontal tDCS - None of the stimulations applied had a positive effect on WM training gains. |
| (Borghini et al., 2018) | - 50 participants (25 elderly and 25 younger adults) - Exp.1: no stimulation - Exp.2: 4 Hz/10 Hz/35Hz or sham tACS, - Exp.3: 10Hz versus sham stimulation | - Bilateral parietal tACS | - Retro-cue WM task | - Parietal alpha-tACS in elderly participants resulted in an increase in target responses compared to sham stimulation. |
| (Feurra et al., 2016) | - Beta, alpha, theta, and gamma-tACS. - 28 participants (young adult 18-35 years) and middle-aged adults (36-60 years) | - Left posterior parietal cortex - Reference electrode: ipsilateral shoulder | - Forward memory span for digit | - Beta-tACS enhanced forward memory span for digits in young adults. |
| (Misselhorn et al., 2020) | - 33 participants - Healthy younger and older adults - 20 Hz beta and 70 Hz gamma-tACS effect on WM outcomes | - Parieto-central | - Visuo-tactile delayed match-to-sample task | - 20 Hz (beta)-tACS speeded the response - 70-Hz (gamma)-tACS delayed the response - The speeding effect of beta-tACS was more evidence in older adults |
| **Cross frequency coupling- tACS** | | | | |
| (Alekseichuk et al., 2016) | 47 participants  Three experimental groups were used: subjects were instructed to complete tasks during the stimulation, first group: sham stimulation, second group: continuous low-frequency theta stimulation, third group: cross-frequency coupling tACS between theta and gamma frequencies | Left prefrontal cortex  Four return electrodes were equally spaced at a distance of 6 cm around the main electrode | Match-to-sample task | The positive effect of continuous low-frequency entrainment on WM performance was abolished by synchronizing high gamma bursts with the troughs of theta cycles.  Significant improvement in WM performance was found when high oscillation gamma bursts (80-100 Hz frequency range) were embedded in the peaks of theta cycles. |
